# Supplementary material for: Cell Type-Specific In Vitro Gene Expression Profiling of Stem Cell-Derived Neural Models
Source: Cells. 2020 Jun 5;9(6):1406. doi: 10.3390/cells9061406 (PMC7349756; doi:10.3390/cells9061406)
Supplement: Supplementary file 1 [file cells-09-01406-s001.zip › supplemental_Figures_EH.docx]

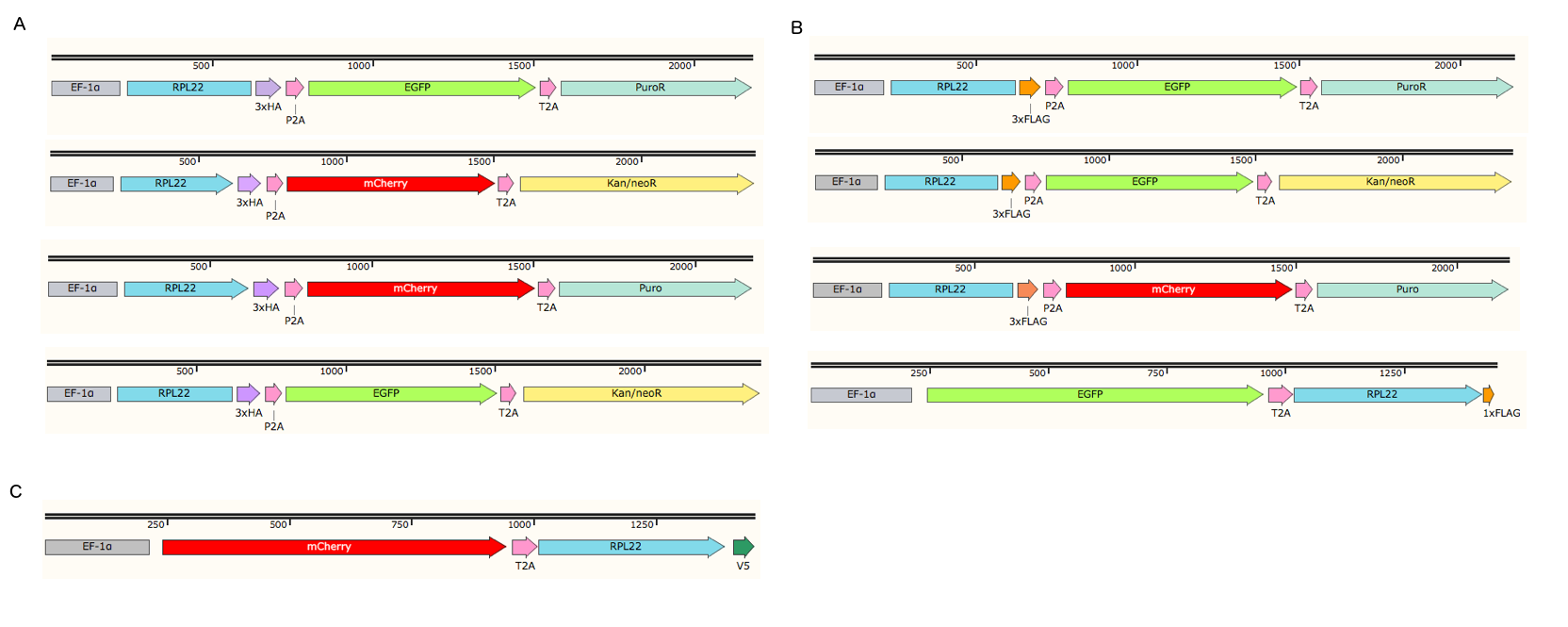


**Figure S1. Diagram of RiboTag lentiviral constructs**. RPL22 was fused to (A) haemagglutinin – HA, (B) 3xFlag or Flag, or (C) V5 epitope tags. RiboTags are expressed as part of a polycistronic mRNA with a combination of fluorescent reporters and/or selectable markers separated by picornavirus 2A peptides.


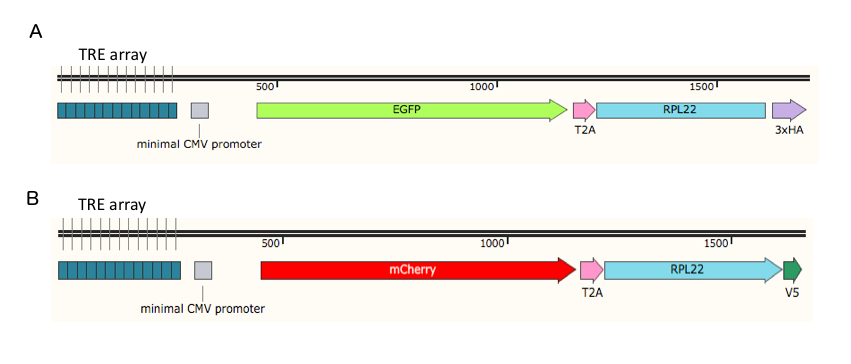


**Figure S2. Diagram of inducible RiboTag lentiviral constructs**. RPL22 was fused to (A) haemagglutinin – HA or (B) V5 epitope tags. RiboTag expression is induced by the addition of doxycycline. Epitope-tagged RPL22 was moved to the 3’ end of the polycistronic mRNA to minimize extra amino acids in the epitope tag. Tetracycline response element (TRE).


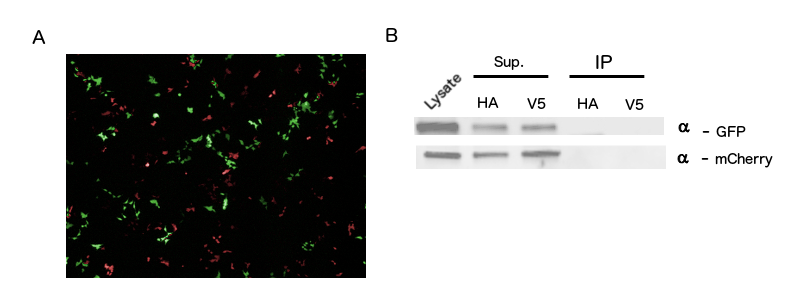


**Figure S3. Co-cultures of HEK293 cells transduced with V5 and HA RiboTag vectors**. GFP and mCherry protein accumulation in HEK293 cells transduced with PTRE-RiboTag lentivirus were confirmed by (A) microscopy and (B) Western blot. Sup – supernatant. IP – immunoprecipitation.


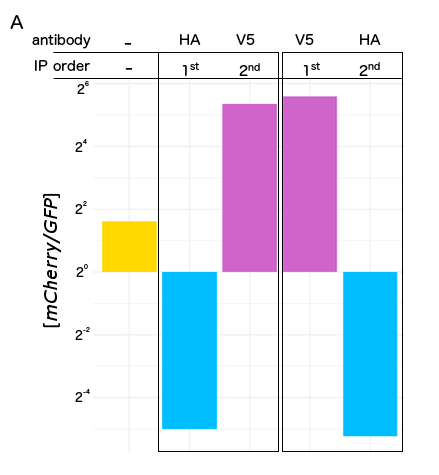


**Figure S4. Reverse transcription digital PCR (RT-dPCR) of *mCherry* and *GFP* from co-cultured human (HEK293) and mouse (NIH-3T3) cells transduced with P_TRE_-GFP-T2A-RPL22-HA and P_TRE_-mCherry-T2A-RPL22-V5, respectively.** The ratio of *mCherry* to *GFP* transcript abundance was measured in triplicate from pre- and post-IP samples and reported as a ratio of the average concentration. IPs were performed in both orders as indicated.


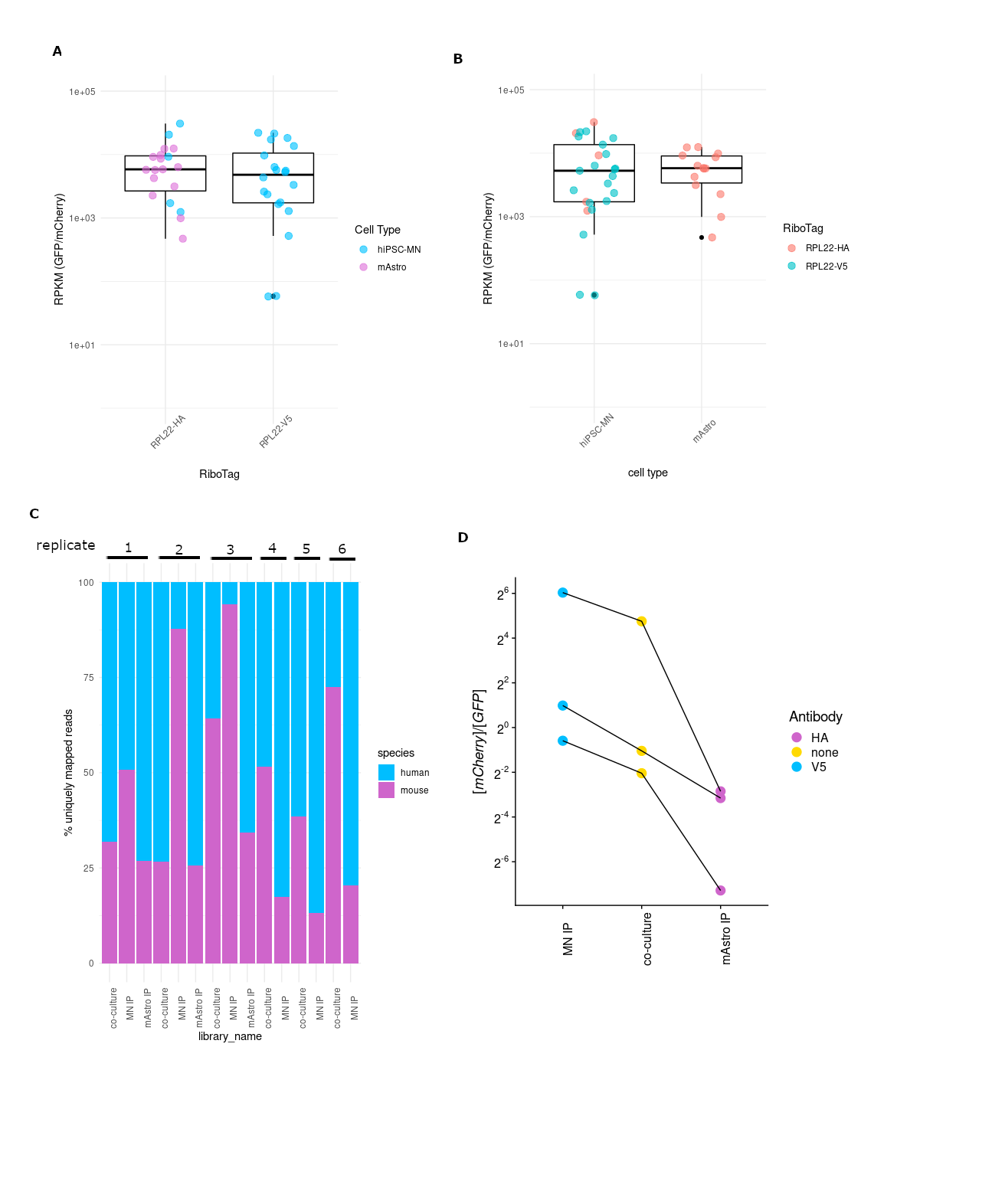


**Figure S5. RiboTag expression and cell-type specific enrichment in hiPSC-MN and primary mouse astrocyte co-cultures**. (A-B) RPKM of reporter gene expression (GFP/mCherry) in primary mouse astrocytes and hiPSC-derived motor neurons split by (A) RiboTag and (B) cell type. (C) RNA-seq reads were mapped to a hybrid reference genome containing hg38 and mm20 chromosomes and quantified by species for co-cultures and IP samples. Matched samples are indicated by replicate number. (D) mCherry and GFP levels were measured by RNA-seq. The ratio of mCherry to GFP is reported for co-cultures and IP samples from hiPSC-MNs (MN IP) and primary mouse astrocytes (mAstro IP). HA RiboTag (purple). V5 RiboTag (blue). Lines indicate matched IPs and co-cultures.


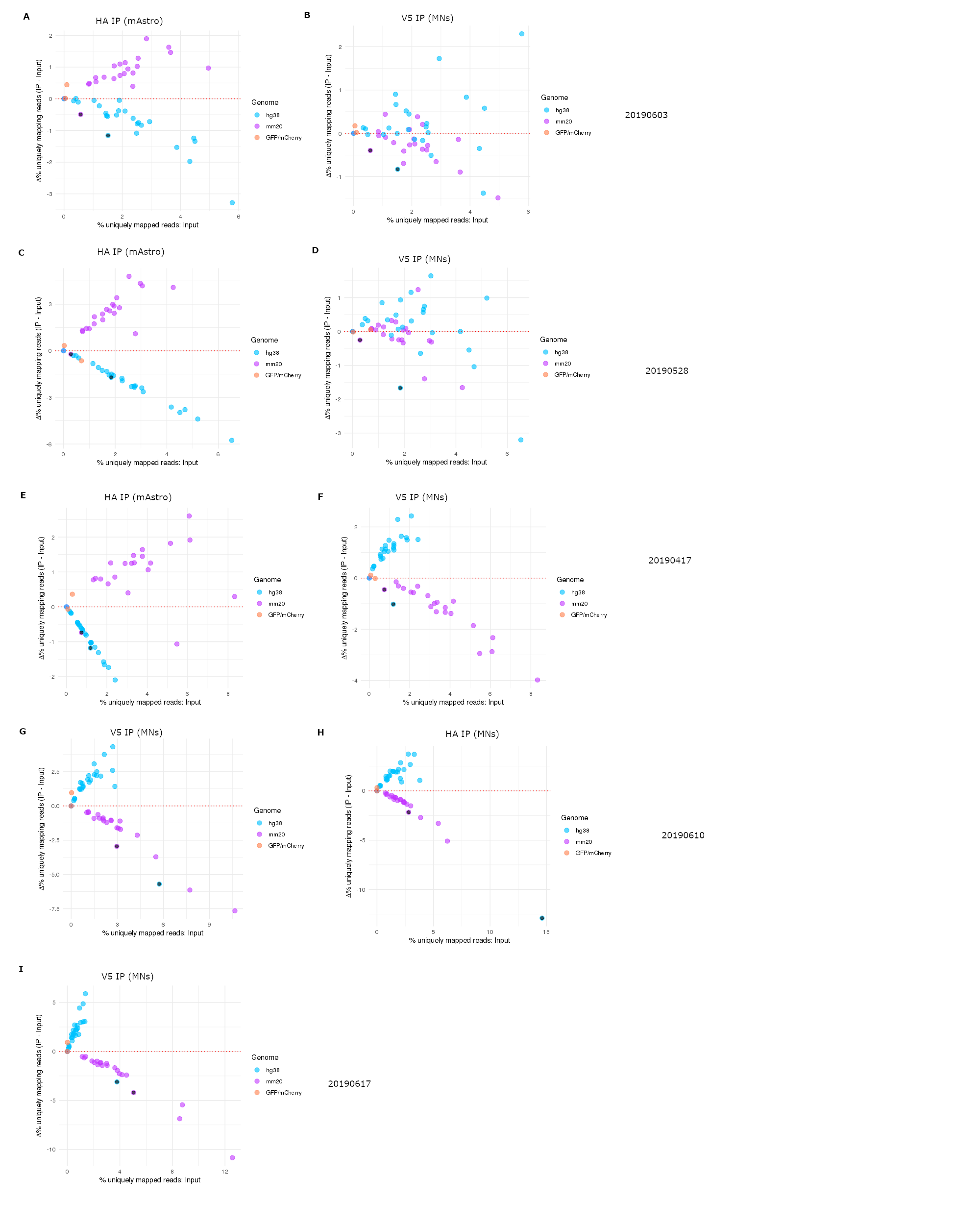


**Figure S6. Relative enrichment of human and mouse RNA in RiboTag IPs compared to co-cultures of hiPSC-MNs and primary mouse astrocytes**. The change in uniquely mapping reads (IP – input) for each chromosome was plotted against the initial co-culture (Input). Each dot represents a chromosome (hg38 – blue, mm20 – purple; mitochondria are indicated with black fill). Chromosomes that fall above the red dotted line are enriched whereas chromosomes that fall below the red dotted line were depleted in IP samples compared to the Input. (A-B) Matched HA and V5 RiboTag IPs from replicate 1. (C-D) Matched HA and V5 RiboTag IPs from replicate 2. (E-F) Matched HA and V5 RiboTag IPs from replicate 3. (G-I) V5 or HA RiboTag IPs for replicates 4, 5, and 6. Target cell type is indicated for each plot.


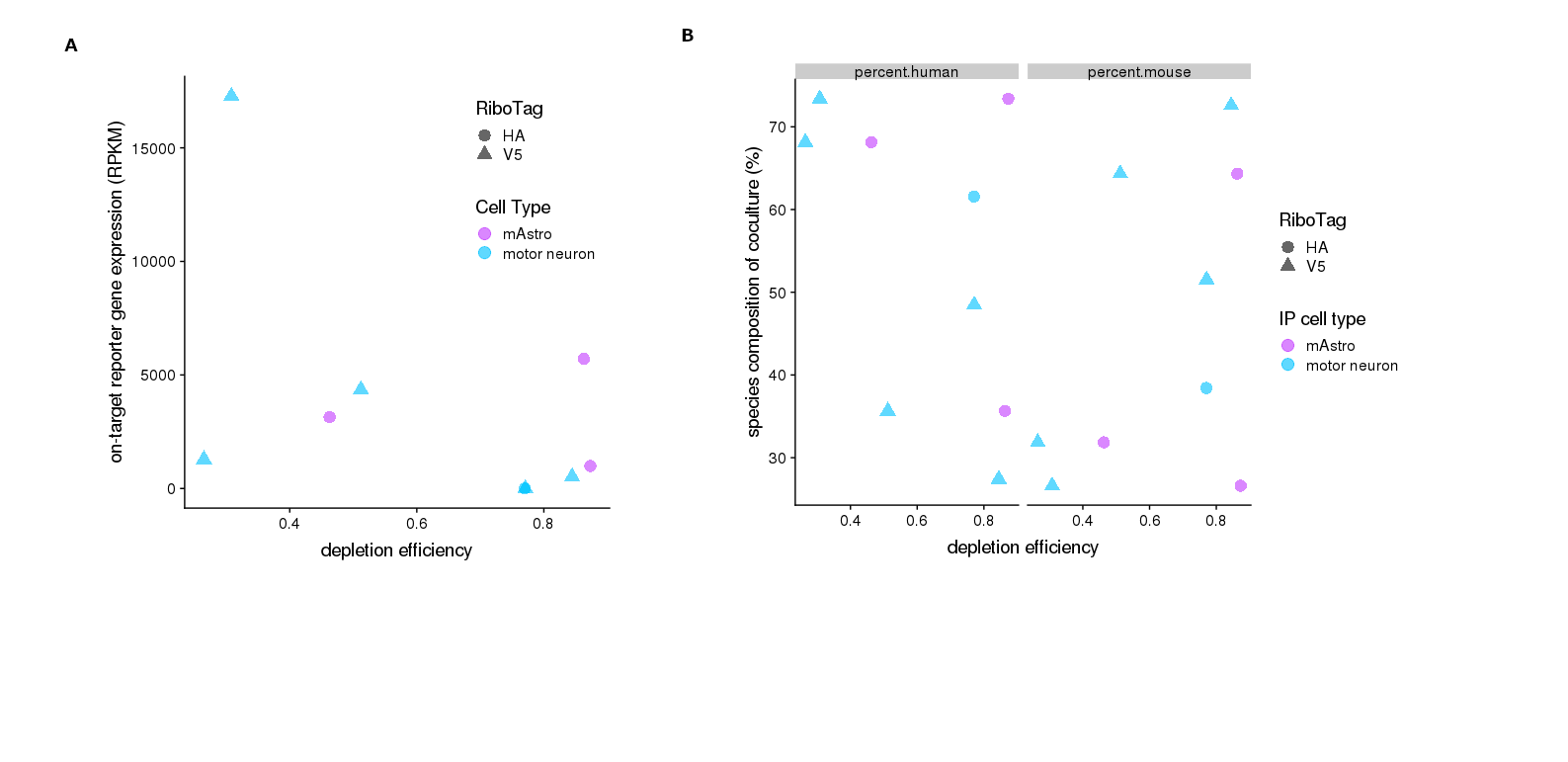


**Figure S7. Correlation of RiboTag expression and the initial coculture species composition with depletion efficiency of RiboTag IPs**. (A) Scatter plot of on-target reporter (RPL22-HA-GFP; RPL22-V5-mCherry) gene expression in RPKM versus depletion efficiency. RPKM was calculated using the library size for the indicated cell type (i.e. only human reads were used to calculate RPKM for reporter expression in hiPSC-MNs and vice versa). (B) Scatter plot of the initial species composition of the coculture (left – human; right - mouse) versus the corresponding depletion efficiency for every IP. The on-target cell type is indicated by color (mAstro – purple; hiPSC-MN – blue) and shape indicates the RiboTag (HA – circles, V5 – triangles). For example, blue circles & triangles on the left examine the relationship between the depletion efficiency and the relative composition of the on-target cell type (human) in the initial coculture. The purple circles on the left examine the relationship between the depletion efficiency and the off-target cell type (mouse). The reverse is true for the plot on the right.


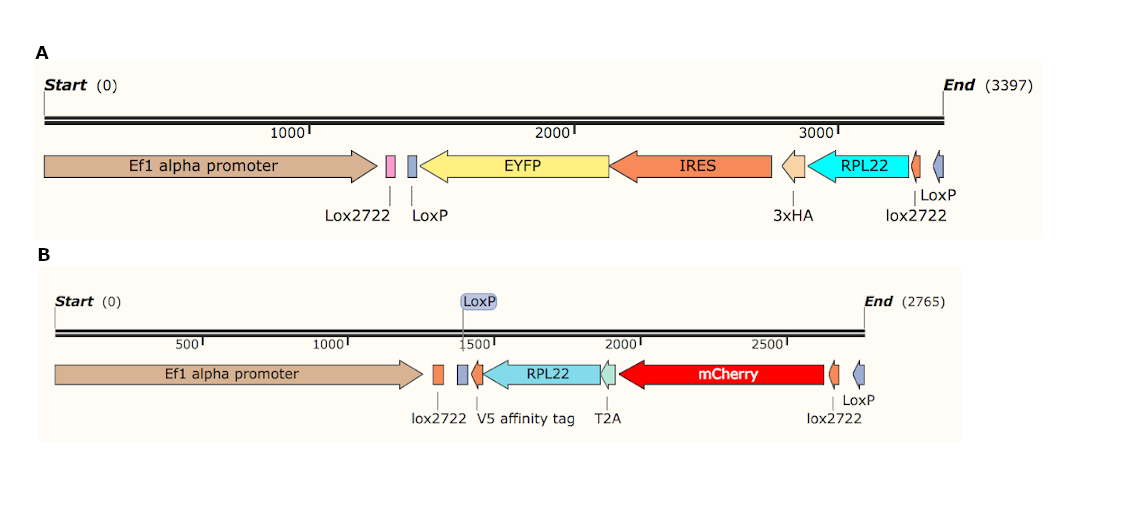


**Figure S8. Diagram of double-floxed inverse orientation (DIO) lentiviral RiboTag constructs.** (A) EFIa-DIO RPL22-IRES-YFP was amplified from pAAV-Ef1a-DIO- Rpl22-3xHA-IRES-eYFP (gift from Stan McKnight) and cloned into existing RiboTag lentiviral vectors (B) DIO mCherry-T2A-RPL22-V5 RiboTag. RiboTag expression is induced in the presence of Cre, which flips the RiboTag orientation to match the Ef1a promoter.


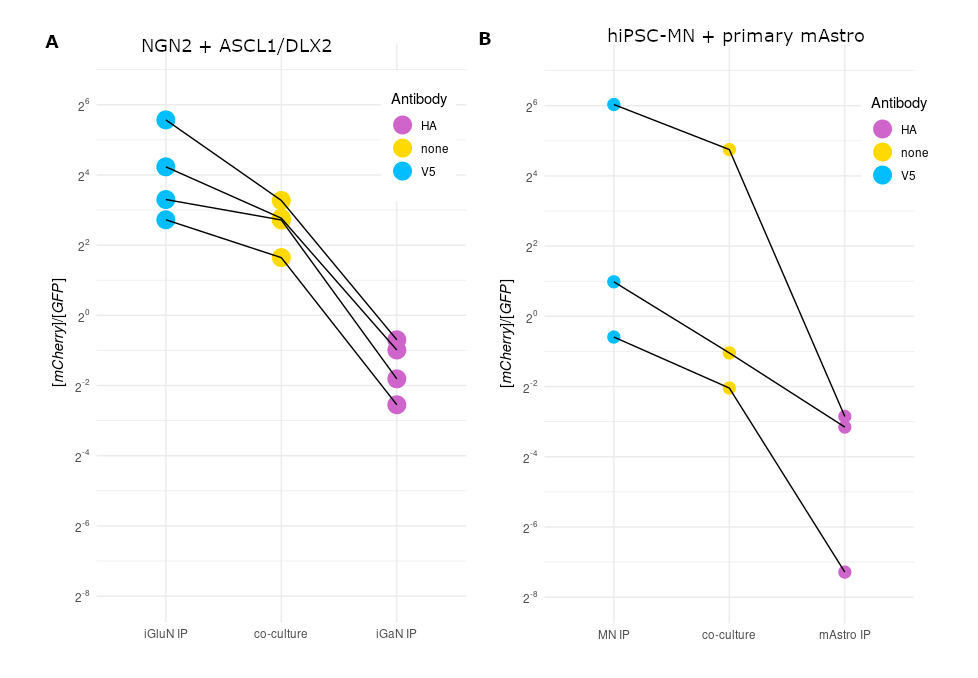


**Figure S9. Reverse transcription digital PCR (RT-dPCR) of mCherry and GFP from RiboTag co-cultures.** Plotted is the ratio of mCherry to GFP across co-cultures (yellow), V5 IPs (blue), and HA IPs (purple) for (A) NGN2-induced excitatory neuron (iGluN) and ASCL1/DLX2-induced GABAergic neuron (iGaNs) co-cultures prepared from a single differentiation and RiboTag transduction per cell type, and (B) primary mouse astrocytes (mAstro) and hiPSC-MN co-cultures across independent replicates. Lines indicated matched IPs and co-cultures.


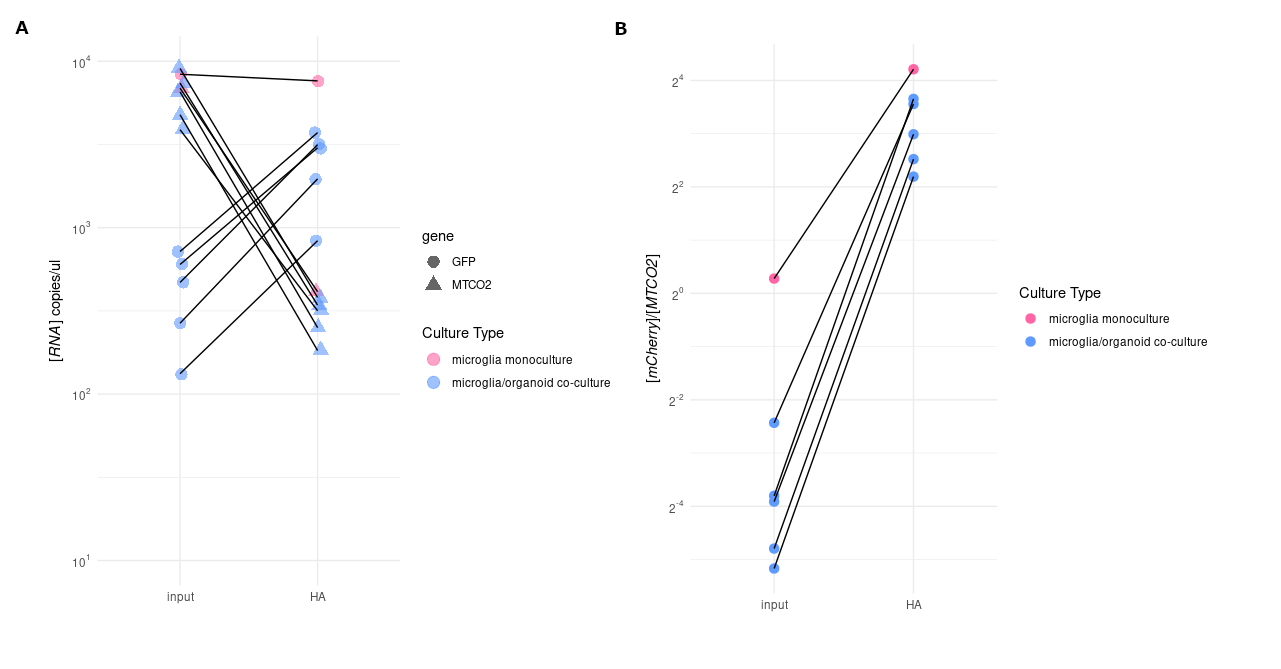


**Figure S10. Reverse transcription digital PCR (RT-dPCR) of GFP and MTCO2 from HMC3 microglia expressing GFP-T2A-RPL22-HA co-cultured with neurospheres**. Input and HA-immunoprecipitated RNA was purified from stable HMC3 microglia grown alone and in co-culture with neural organoids. GFP and MTCO2 were measured by RT-dPCR and (A) plotted as absolute RNA levels and (B) a ratio of GFP to MTCO2. Lines indicate matched input and IPed samples.
